# Supplementary material for: The Di-Symbiotic Systems in the Aphids Sipha maydis and Periphyllus lyropictus Provide a Contrasting Picture of Recent Co-Obligate Nutritional Endosymbiosis in Aphids
Source: Microorganisms. 2022 Jul 6;10(7):1360. doi: 10.3390/microorganisms10071360 (PMC9317480; doi:10.3390/microorganisms10071360)

Figure S1. Geographic location of the collection sites of *S. maydis* colonies assayed in this study (for details, see Table S1). Green spots reference the 2014 sampling and red spots reference the 2016 sampling.

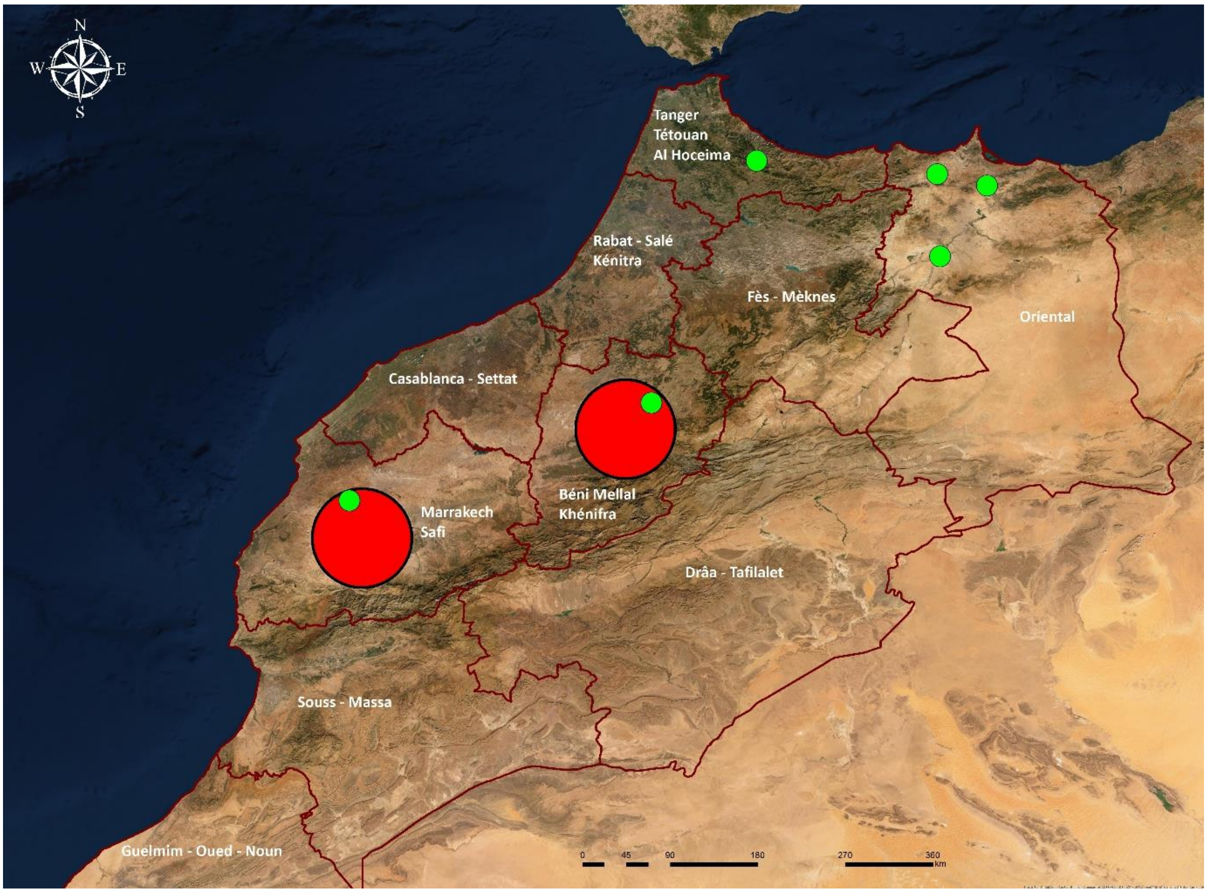

Supplement: Supplementary file 1 [file microorganisms-10-01360-s001.zip › microorganisms-1791456-supplementary/Figure S1.pdf]
